# Confirming SPSS Results With ChatGPT-4 and o3-mini Models

**DOI:** 10.7759/cureus.82005

**Published:** 2025-04-10

**Authors:** Frederick Strale, Isaac Riddle, Bowen Geng, Blake Oxford, Malia Kah, Robert Sherwin

**Affiliations:** 1 Biostatistics, The Oxford Center, Brighton, USA; 2 Information Technology, The Oxford Center, Brighton, USA; 3 Research, The Oxford Center, Brighton, USA; 4 Hyperbaric Oxygen Therapy, Wayne State University School of Medicine, Detroit, USA

**Keywords:** artificial intelligence in scientific writing, chatgpt, chatgpt 4, chatgpt o3-mini-model, openai, statistical analysis (spss)

## Abstract

Background

This research compared the simple and advanced statistical results of SPSS (IBM Corp., Armonk, NY, USA) with ChatGPT-4 and ChatGPT o3-mini (OpenAI, San Francisco, CA, USA) in statistical data output and interpretation with behavioral healthcare data. It evaluated their methodological approaches, quantitative performance, interpretability, adaptability, ethical considerations, and future trends.

Methods

Fourteen statistical analyses were conducted from two real datasets that produced peer-reviewed, published scientific articles in 2024. Descriptive statistics, Pearson r, multiple correlation with Pearson r, Spearman's rho, simple linear regression, one-sample t-test, paired t-test, two-independent sample t-test, multiple linear regression, one-way analysis of variance (ANOVA), repeated measures ANOVA, two-way (factorial) ANOVA, and multivariate ANOVA were computed. The two datasets adhered to a systematically structured timeframe, March 19, 2023, through June 11, 2023, and June 7, 2023, through July 7, 2023, thereby ensuring the integrity and temporal representativeness of the data gathering. The analyses were conducted by inputting the verbal (text) commands into ChatGPT-4 and ChatGPT o3-mini along with the relevant SPSS variables, which were copied and pasted from the SPSS datasets.

Results

The study found high concordance between SPSS and ChatGPT-4 in fundamental statistical analyses, such as measures of central tendency, variability, and simple Pearson and Spearman correlation analyses, where the results were nearly identical. ChatGPT-4 also closely matched SPSS in the three t-tests and simple linear regression, with minimal effect size variations. Discrepancies emerged in complex analyses. ChatGPT o3-mini showed inflated correlation values and significant results where none were expected, indicating computational deviations. ChatGPT o3-mini produced inflated coefficients in the multiple correlation and R-squared values in two-way ANOVA and multiple regression, suggesting differing assumptions. ChatGPT-4 and ChatGPT o3-mini produced identical F-statistics with repeated measures ANOVA but reported incorrect degrees of freedom (df) values. While ChatGPT-4 performed well in one-way ANOVA, it miscalculated degrees of freedom in multivariate ANOVA (MANOVA), leading to significant discrepancies. ChatGPT o3-mini also generated erroneous F-statistics in factorial ANOVA, highlighting the need for further optimization in multivariate statistical modeling.

Conclusions

This study underscored the rapid advancements in artificial intelligence (AI)-driven statistical analyses while highlighting areas that require further refinement. ChatGPT-4 accurately executed fundamental statistical tests, closely matching SPSS. However, its reliability diminished in more advanced statistical procedures, requiring further validation. ChatGPT o3-mini, while optimized for Science, Technology, Engineering, and Mathematics (STEM) applications, produced inconsistencies in correlation and multivariate analyses, limiting its dependability for complex research applications. Ensuring its alignment with established statistical methodologies will be essential for widespread scientific research adoption as AI evolves.

## Introduction

The past two decades have witnessed unprecedented advancements in science, medicine, and technology, significantly enhancing human life and efficiency. Among these breakthroughs, artificial intelligence (AI) has emerged as a transformative force, surpassing previous cognitive reasoning, problem-solving, and automation innovations. OpenAI’s ChatGPT (San Francisco, CA, USA), a state-of-the-art language model, has been particularly influential, demonstrating sophisticated natural language processing capabilities applicable across multiple domains, including education, business, and healthcare [1]. In medicine, AI-driven models like ChatGPT are being integrated into clinical workflows for drug testing, diagnostic imaging, and precision medicine, reshaping the landscape of modern healthcare [1-5].

Statistical data analysis is crucial in medical and epidemiological research, where accuracy, transparency, and methodological rigor are paramount. Traditionally, statistical software such as IBM’s Statistical Package for the Social Sciences (SPSS; IBM Corp., Armonk, NY, USA) has been widely employed for quantitative analysis due to its structured, deterministic approach. SPSS provides a user-friendly interface, enabling researchers to conduct various statistical tests, including t-tests, analysis of variance (ANOVA), regression, and correlation analyses, while ensuring methodological transparency and reproducibility [6-8]. However, recent developments in AI-powered tools, particularly ChatGPT-4 and its current successor, ChatGPT o3-mini, have introduced new avenues for performing statistical computations with enhanced flexibility and automation [6].

ChatGPT-4, leveraging Python libraries such as NumPy, Pandas, and SciPy, has demonstrated the capability to execute a range of statistical analyses, from descriptive statistics to more advanced inferential techniques [6]. Unlike SPSS, which follows a procedural, menu-driven workflow, ChatGPT-4 operates interactively through natural language queries, offering a dynamic and adaptive analytical experience. Despite these advantages, concerns persist regarding its consistency, transparency, and precision compared to established software like SPSS [8-13].

The recent release of ChatGPT o3-mini in January 2025 marks a pivotal evolution in AI-driven statistical analysis. Designed to enhance performance in Science, Technology, Engineering, and Mathematics (STEM) disciplines, including advanced mathematical and statistical reasoning, ChatGPT o3-mini balances computational efficiency and cost-effectiveness. However, coding enhancements remain necessary to minimize technical errors and ensure strong performance across diverse datasets [7,13].

While prior research has explored ChatGPT-4’s effectiveness in executing fundamental statistical tests such as two-independent sample t-tests, chi-square tests, and simple linear regression, its capability to perform more sophisticated multivariate and inferential analyses has mainly been unexamined [1,6,8]. Moreover, comparative studies assessing ChatGPT-based models alongside SPSS for statistical analysis in medical research are still limited. The introduction of ChatGPT o3-mini, with its refined computational logic and enhanced reasoning ability, presents a compelling opportunity to reevaluate AI-driven statistical methodologies compared to traditional software solutions [14-17].

Study objective and rationale

The primary aim of this study is to build upon prior research, systematically assessing and comparing the concordance of SPSS, ChatGPT-4, and ChatGPT o3-mini in executing simple and complex statistical procedures commonly utilized in medical and epidemiological research [1,6,8]. By examining their methodological approaches, quantitative accuracy, interpretability, and adaptability, this research aims to evaluate AI-assisted statistical tools comprehensively. 

Our rationale is to address the existing knowledge gap by investigating the performance of AI-driven statistical platforms compared to traditional statistical platforms such as SPSS. By doing so, we aim to provide insights that will inform best practices for integrating AI into biomedical research.

Our authors are motivated to understand how AI tools like ChatGPT-4o and ChatGPT 3o-mini compare to traditional statistical software like SPSS in terms of accuracy, efficiency, and overall performance. As AI continues to evolve and become more sophisticated, evaluating its potential to enhance or transform the way statistical analyses are conducted in biomedical research is crucial.

By testing these AI-driven platforms, we seek to determine their strengths and limitations in handling simple and complex statistical tasks. This includes assessing their ability to perform advanced multivariate and inferential analyses, which is essential for drawing meaningful conclusions from biomedical data.

Ultimately, our study intends to contribute to developing best practices for integrating AI into biomedical research. By evaluating AI-driven statistical methods, we aim to guide researchers in making informed decisions about the tools they use for data analysis. This will help ensure that AI is used effectively and responsibly, maximizing its potential to advance scientific knowledge and improve health outcomes.

## Materials and methods

Data collection 

This study integrates and critically evaluates two quantitative datasets from an analysis of peer-reviewed and published studies conducted at The Oxford Center in Brighton, Michigan. Two real datasets adhered to a systematically structured timeframe, March 19, 2023, through June 11, 2023 [18], and June 7, 2023, through July 7, 2023 [19], thereby ensuring the integrity and temporal representativeness of the datasets. The analyzed data primarily examined the efficacy of applied behavior analysis (ABA) in modifying targeted behaviors among autistic individuals, contributing to the broader discourse on evidence-based therapeutic interventions. 

ABA data were selected for analysis due to their methodological suitability for addressing the original papers' research objectives. The datasets delineated independent variables appropriate for group comparisons, such as intervention phases or treatment conditions, and dependent variables that captured quantifiable behavioral outcomes. Importantly, the repeated measures structure of the data facilitated within-subject comparisons over time, thereby increasing statistical power and enabling the examination of treatment effects across multiple observation points.

The datasets and corresponding statistical results were contained within the SPSS 29.0 research database at The Oxford Center for comparative analysis of SPSS results with ChatGPT outputs. Each statistical test was executed per the appropriate analytical procedures within SPSS, ensuring adherence to established methodological protocols and best practices. To maintain the integrity and validity of the comparative analysis, identical datasets were utilized for both SPSS, ChatGPT-4, and ChatGPT o3-mini without any modifications, transformations, or preprocessing alterations post-import. This methodological consistency ensured that both platforms operated under equivalent analytical conditions. 

The datasets were initially extracted from the "Catalyst" commercial electronic data collection tool, which is widely used for applied behavior analysis. These datasets were then exported into SPSS for subsequent analysis. Data accuracy was ensured by logic checks and consistent formatting to maintain uniformity, i.e., numeric variables were kept separate from categorical (grouping) variables, ensuring that each type was processed correctly. Clear labeling provided context, making it easier to interpret results. Our research utilized historical SPSS data to compare their outputs with ChatGPT-4o and ChatGPT o3-mini.

Given that any error detection issues were addressed and resolved during the initial SPSS data entry, handling, and logic checks, the datasets used in this study were already validated and accurate. This ensured that the comparisons between SPSS, ChatGPT-4o, and ChatGPT o3-mini were based on high-quality data, enhancing our findings' credibility.

Data prompts for ChatGPT-4o and ChatGPT o3-mini

The following analyses were conducted by inputting the verbal (text) prompts below and the relevant SPSS variables, which were copied from the SPSS dataset and pasted into ChatGPT-4 and ChatGPT o3-mini. Please see Table 1 below.

**Table 1:** Test statistic with ChatGPT-4 and ChatGPT o3-mini data prompts

Test Statistic	Data Prompts for ChatGPT-4 & ChatGPT o3-mini
Descriptive Statistics	"Please compute the mean, standard deviation, median, and mode for the variables Age and Time1 in this SPSS dataset."
Pearson correlations (r)	"Please compute the Pearson r (with confidence interval) with p-values for the variables Age and Time1 in this SPSS dataset."
Multiple Correlations (r)	"Please produce a Pearson r Correlation matrix for this SPSS dataset with the variables Age, Time1, Time2, and Time3. Please include confidence intervals and p-values (two-tailed)."
Spearman's Rho	"Please produce a Spearman's Rho for this SPSS dataset with the variables Age and Time1."
Spearman's Rho Multiple Correlations	"Please produce a Spearman's rho Correlation matrix for this SPSS dataset with the variables Age, Time1, Time2, and Time3. Please include confidence intervals and p-values (two-tailed)."
Simple Linear Regression	"Please run a simple linear regression with these SPSS variables, Time2 as the predictor (independent) variable and Time3 as the outcome (dependent) variable. Please compute R, R^2^, adjusted R^2^, Standard Error of the Estimate, F, p-value, t, p-value, B, Standard Error, and Beta."
One Sample t-test	"Please run a One Sample t-test with mu=0, with this Time1 variable from SPSS. Please compute the n, mean, standard deviation, standard error of the mean, t, df, p-value, Mean difference, 95% confidence interval, Cohen's d, and the 95% confidence interval for Cohen's d."
Paired t-test	"Please run a Paired t-test, with this Time1 variable as the Pretest and the Time3 variable as the Posttest from SPSS. Please compute the n, Pretest Mean, Posttest Mean, standard deviation, standard error of the mean, t, df, p-value, Mean difference, 95% confidence interval, Cohen's d, and the 95% confidence interval for Cohen's d."
Two-Independent Sample t-test	"Please run a Two Independent Sample t-test, with gender as the grouping variable (independent variable) and Time3 as the dependent variable with this SPSS dataset. Please compute the n, the Group 1 and Group 2 Means, standard deviations, standard errors of the means, t, df, p-value, Mean difference, 95% confidence interval, Cohen's d, and the 95% confidence interval for Cohen's d."
Multiple Linear Regression	"Please run a multiple linear regression with these SPSS variables, Age, Time1, and Time2 as the predictor (independent) variables and Time3 as the outcome (dependent) variable. Please compute R, R^2^, adjusted R^2^, Standard Error of the Estimate, F, p-value, t, p-value, B, Standard Error, and Beta."
One-Way Analysis of Variance (ANOVA)	"Please run a One-Way Analysis of Variance (ANOVA) with Age Category as the Grouping (independent variable) with five groups and Time3 as the dependent variable. Please compute the "ANOVA Table" with Between and Within Groups Sums of Squares and Between and Within Groups Mean Square, F, and p-value."
Repeated Measures ANOVA	"Please run a Repeated Measures ANOVA on these SPSS Time1, Time2, and Time3 variables. Targets Mastered Baseline, Targets Mastered 2 Weeks, and Targets Mastered 4 Weeks."
Two-Way (Factorial) ANOVA	"Please run a Two-Way (Factorial) Analysis of Variance (ANOVA) with Gender (2 groups) and AgeCategory (5 groups) as the Grouping (independent) variables and Time3 as the dependent variable. Please compute the "ANOVA Table" with Source (Corrected Model, Intercept, Gender, AgeCategory, Gender x AgeCategory, Error, Total, and Corrected Total), with Type III Sum of Squares, df, Mean Square, F, and p-values. Also, please do an Adjusted R-squared computation."
Multivariate Analysis of Variance (MANOVA)	"Please run a Multivariate Analysis of Variance (MANOVA) with this SPSS dataset using Gender as the independent (grouping) variable, and AvgTrialsToMastery, AvgTeachingdaysToMastery, OpenTargets, PercentofTargetsMastered, PercentofTargetsFailedin Maintenance, PercentofTargetsTrendingUp, PercentofTargetsTrendingDown, PercentofTargetsTrendingFlat, as the dependent variables. Please run Box's M and other tests of assumptions and report the Multivariate Tests' relevant statistics for Pillai's Trace, Wilks' Lambda, Hotelling's Trace, and Roy's Largest Root. Gender AvgTrialsToMastery, AvgTeachingDaysToMastery, OpenTargets, PercentofTargetsMastered, PercentofTargetsFailedinMaintenance, PercentofTargetsTrendingUp, PercentofTargetsTrendingDown, PercentofTargetsTrendingFlat."

Strict attention was given to data formatting within SPSS to ensure consistency in variable classifications (e.g., numeric versus categorical variables), thereby preserving uniform input structures across the three platforms. By maintaining these stringent controls, the study facilitated a direct, objective comparison of the outputs generated by SPSS, ChatGPT-4, and ChatGPT o3-mini, thereby ensuring methodological rigor and the comparability of results across both analytical platforms. 

Simple statistical analyses

This category comprises foundational techniques that summarize datasets and provide preliminary insights into their central tendency and variability. These methods are essential for understanding the data's distribution and characteristics prior to applying more complex inferential procedures.

Mean

The mean represents the arithmetic average of all values in a dataset, serving as a measure of central tendency. It is sensitive to outliers, which can disproportionately influence its value.

Standard Deviation (SD)

SD quantifies the dispersion of data points around the mean. A smaller SD indicates that values are closely clustered around the mean, while a larger SD reflects greater variability within the dataset.

Median

The median is the midpoint value when all observations are ordered numerically. Unlike the mean, it is robust to outliers and skewed distributions, making it a useful measure when data are not normally distributed*.*

Mode

The mode identifies the most frequently occurring value in a dataset. It is particularly useful for categorical or discrete variables and for understanding the shape of the distribution, including the presence of multimodal trends.

Inferential statistics with hypothesis testing

These analyses allow researchers to test hypotheses about relationships between variables or differences between groups, and to generalize findings from a sample to a broader population.

Pearson Correlation Coefficient (r)

Pearson r assesses the strength and direction of the linear relationship between two continuous variables. Values range from -1 to +1, with values near ±1 indicating strong linear relationships. This test assumes both variables are approximately normally distributed and measured at the interval or ratio scale.

Spearman Rank-Order Correlation (Rho)

Spearman’s ρ is a non-parametric alternative to Pearson’s r, used when data are ordinal or when assumptions of normality are violated. It evaluates whether the relationship between variables is monotonic (i.e., consistently increasing or decreasing), without assuming linearity.

Simple Linear Regression

This technique predicts the value of a continuous dependent variable based on a single independent (predictor) variable. It estimates the strength of the linear association and provides coefficients that quantify the expected change in the dependent variable for each unit change in the predictor.

One-Sample T-Test

This test evaluates whether the mean of a single sample differs significantly from a known or hypothesized population mean. It is commonly used to assess whether a treatment effect or observed outcome departs meaningfully from an expected baseline.

Paired Sample T-Test

The paired t-test compares two related means, such as measurements taken from the same subjects before and after an intervention. It accounts for the within-subject variability and is useful for detecting changes over time or across conditions in repeated measures designs.

Two-Independent Sample T-Test

This test compares the means of two independent groups to determine whether there is statistical evidence that the associated population means are significantly different. It assumes that the data are normally distributed and that the variances of the two groups are approximately equal.

Advanced statistical analyses

This section includes multivariate and factorial methods capable of modeling more complex relationships among variables. These approaches are particularly useful when dealing with multiple predictors or dependent variables, and when evaluating interactions.

Multiple Linear Regression

An extension of simple linear regression, this technique estimates the relationship between one continuous dependent variable and two or more independent variables. It enables the examination of unique contributions of each predictor while controlling for the influence of others, and is frequently used to assess confounding and mediating effects.

One-Way ANOVA

This test determines whether there are statistically significant differences among the means of three or more independent groups. It evaluates the between-group variance relative to within-group variance and assumes normal distribution and homogeneity of variances.

Repeated Measures ANOVA

This technique analyzes data collected from the same subjects at multiple time points or under different conditions. It accounts for the correlation between repeated observations and tests whether mean changes over time or across conditions are statistically significant.

Two-Way (Factorial) ANOVA

This method evaluates the independent and interactive effects of two categorical independent variables on a single continuous dependent variable. It allows researchers to investigate not only main effects but also interaction effects, which reveal whether the influence of one factor depends on the level of the other.

Multivariate ANOVA (MANOVA)

MANOVA is an extension of ANOVA used when there are two or more dependent variables. It tests whether the mean vectors of the groups differ across a combination of dependent variables, taking into account the potential correlations among them. MANOVA provides a more holistic view of group differences and can reduce the risk of Type I error when multiple outcomes are assessed simultaneously.

Ethical approval

This research study was conducted retrospectively, using data collected from March 19, 2023, through June 11, 2023 [18], and June 7, 2023, through July 7, 2023 [19], used in peer-reviewed, published studies and chart reviews for clinical purposes. The study was submitted to the Western Copernicus Ethical Approval Group-Institutional Review Board (WCG-IRB) for review and received an exemption (#1-1703366-1). The authors certify that the analysis was performed per the ethical standards as put forth in the 1964 Declaration of Helsinki and its later amendments or comparable ethical standards.

## Results

Simple statistics

Descriptive Statistics

Table 2 below shows that all three analyses for the variable "Age," using SPSS, ChatGPT-4, and ChatGPT o3-mini, yielded identical descriptive statistics. Specifically, the mean age was 9, the SD was 8.15, the median age was 7.5, and the mode was 5. This consistency indicated that the computed central tendency and dispersion measured are the same regardless of the software or model used. The mean of 9 and a median of 7.5 suggested that the distribution might be slightly right-skewed (since the mean was higher than the median), while the mode of 5 indicated that the most common age in the dataset was five.

**Table 2:** Descriptive statistics SD=Standard deviation Variable=Age

Model	Mean	SD	Median	Mode
SPSS	9	8.15	7.5	5
ChatGPT-4	9	8.15	7.5	5
ChatGPT o3-mini	9	8.15	7.5	5

Table 3 below indicates that all three methods, SPSS, ChatGPT-4, and ChatGPT o3-mini, yielded nearly identical results when analyzing the relationship between Age (X) and Time1 (Y). With the descriptive statistics, for X (Age), the mean was consistently 9 with a standard deviation of approximately 8.14-8.15. The mean for Y (Time1) was 5.32, with a standard deviation of 8.98.

**Table 3:** Pearson correlations (r) X=Age, Y=Time, 95%CI=95% Confidence Interval Pearson Product Moment Correlation Coefficient (Pearson r) was used as the statistical test to compute the p-values for this table.

Model	Mean	SD	r (95%CI)	p-value
SPSS	-	-	-	-
X	9	8.14	0.111 (-0.105, 0.317)	0.311
Y	5.32	8.98	-	-
ChatGPT-4	-	-	-	-
X	9	8.15	0.111 (-0.105, 0.317)	0.311
Y	5.32	8.98	-	-
ChatGPT o3-mini	-	-	-	-
X	9	8.14	0.111 (-0.105, 0.317)	0.311
Y	5.32	8.98	-	-

With the correlational analysis, the correlation coefficient (r) between Age and Time1 was 0.111 across all three methods, with a 95% confidence interval from -0.105 to 0.317. The p-value of 0.311 indicated that the correlation was not statistically significant, suggesting no firm evidence of a linear relationship between Age and Time1 in the sample.

The consistency in results across SPSS, ChatGPT-4, and ChatGPT o3-mini reinforced the reliability of the analyses and the negligible standard deviation difference (8.14 vs. 8.15) was most likely due to rounding.

Table 4 below presents Pearson correlation coefficients, p-values, and confidence intervals for different variable pairs, comparing results from SPSS, ChatGPT-4, and ChatGPT o3-mini. SPSS and ChatGPT-4 results were identical. Both showed weak correlations between Age and Time variables (r ranging from 0.111 to 0.159), with non-significant p-values (p > 0.05), suggesting no strong relationship between Age and Time measures. Strong, significant correlations (p < 0.001) were found between Time1, Time2, and Time3, with values above 0.8, indicating high interdependence.

**Table 4:** Multiple Correlations (Pearson r) CI=Confidence Interval Variables=Age, Time1, Time2, Time3, 95%CI=95% Confidence Interval Pearson Product Moment Correlation Coefficient (Pearson r) was used as the statistical test to compute the p-values for this table.

Model	Pearson r	p-value (2-tailed)	95% CI (2-tailed)
SPSS	-	-	-
Age - Time1	0.111	0.311	-0.105, 0.317
Age - Time2	0.156	0.155	-0.059, 0.357
Age - Time3	0.159	0.147	-0.056, 0.359
Time1-Time2	0.872	<0.001	0.813, 0.914
Time1-Time3	0.827	<0.001	0.75, 0.882
Time2-Time3	0.977	<0.001	0.966, 0.985
ChatGPT-4	Pearson r	p-value (2-tailed)	95% CI (2-tailed)
Age - Time1	0.111	0.311	-0.105, 0.317
Age - Time2	0.156	0.155	-0.059, 0.357
Age - Time3	0.159	0.147	-0.056, 0.359
Time1-Time2	0.872	<0.001	0.813, 0.914
Time1-Time3	0.827	<0.001	0.750, 0.882
Time2-Time3	0.977	<0.001	0.966, 0.985
ChatGPT o3-mini	Pearson r	p-value (2-tailed)	95% CI (2-tailed)
Age - Time1	0.317	<0.001	0.073, 0.514
Age - Time2	0.357	<0.001	0.120, 0.545
Age - Time3	0.360	<0.001	0.123, 0.549
Time1-Time2	0.914	<0.001	0.866, 0.942
Time1-Time3	0.882	<0.001	0.823, 0.923
Time2-Time3	0.985	<0.001	0.975, 0.990

ChatGPT o3-mini showed higher correlations and significant p-values. The correlations between Age and Time variables were noticeably higher (r ranging from 0.317 to 0.36) and statistically significant (p < 0.001), which contradicted SPSS and ChatGPT-4 results. The confidence intervals for these correlations were also shifted higher, suggesting a stronger relationship. The correlations between Time variables (Time1-Time2, Time1-Time3, Time2-Time3) were slightly higher than in SPSS and ChatGPT-4 but remained in a similar range (0.914-0.985).

Since SPSS and ChatGPT-4 provided identical results, it suggested they aligned with standard statistical computations. ChatGPT o3-mini showed inflated correlation values and significant p-values, which could indicate differences in calculation methods, rounding, or data handling. Given that the dataset is likely the same across tools, the discrepancies in o3-mini’s results might stem from a different statistical approach or computational variation in its implementation.

SPSS and ChatGPT-4 results appeared more consistent and in line with traditional statistical analysis. ChatGPT o3-mini may have overestimated relationships between Age and Time variables, warranting caution when interpreting its results. If accuracy is critical, verification using SPSS or ChatGPT-4 is recommended.

Table 5 below reports descriptive statistics (mean and SD) and Spearman correlation results (r_sp_) for the variables X (Age) and Y (Time1) across the three SPSS, ChatGPT-4, and ChatGPT o3-mini models. The descriptive statistics were consistent, with the mean and standard deviation for X (Age) and Y (Time1) identical across all three models. The mean for X=9, SD for X: ~8.14-8.15. The mean for Y=5.32, with the SD for Y equal to 8.98. The correlation results were nearly identical. The r_sp_ was 0.058 across all models, indicating a very weak relationship between Age and Time1. The p-value (0.595) was non-significant, suggesting no meaningful correlation. Confidence intervals (95% CI) were almost the same across models, with only slight variation in lower and upper bounds.

**Table 5:** Spearman's Rho X=Age, Y=Time1, SD=Standard deviation, 95%CI=95% Confidence Interval Spearman's Rho was used as the statistical test to compute the p-values for this table.

Model	Mean	SD	Spearman's Rho (95%CI)	p-value (2-tailed)
SPSS	-	-	-	-
X	9	8.14	0.058 (-0.163, 0.274)	0.595
Y	5.32	8.98	-	-
ChatGPT-4	-	-	-	-
X	9	8.15	0.058 (-0.157, 0.268)	0.595
Y	5.32	8.98	-	-
ChatGPT o3-mini	-	-	-	-
X	9	8.14	0.058 (-0.157, 0.268)	0.595
Y	5.32	8.98	-	-

With comparison of the models SPSS vs. ChatGPT-4, the results were nearly identical, with a tiny difference in confidence intervals (-0.163 to 0.274 in SPSS vs. -0.157 to 0.268 in ChatGPT-4). With SPSS vs. ChatGPT o3-mini, identical to ChatGPT-4, meaning both AI models provided results that closely aligned with traditional SPSS statistical software.

The findings suggested no significant correlation between Age and Time1. ChatGPT-4 and ChatGPT o3-mini produced results nearly identical to SPSS, with only minor differences in confidence intervals. These small variations were negligible and did not impact the overall interpretation, confirming that all three models produced statistically equivalent results.

Table 6 below presents Spearman’s rank-order multiple correlation results across SPSS, ChatGPT-4, and ChatGPT o3-mini, including Spearman correlation coefficients, p-values, and confidence intervals. SPSS and ChatGPT-4 results were nearly identical. Both models showed very weak and non-significant correlations between Age and Time variables (correlation values close to zero, p-values > 0.05).

**Table 6:** Spearman's Rho Multiple Correlations 95%CI=95% Confidence Interval Spearman's Rho was used as the statistical test to compute the p-values for this table.

Model	Spearman's Correlation	p-value (2-tailed)	95% CI (2-tailed)
SPSS	-	-	-
Age - Time1	0.058	0.595	-0.163, 0.274
Age - Time2	-0.012	0.910	-0.231, 0.207
Age - Time3	0.012	0.911	-0.208, 0.231
Time1-Time2	0.752	<0.001	0.644, 0.831
Time1-Time3	0.629	<0.001	0.483, 0.741
Time2-Time3	0.911	<0.001	0.867, 0.941
Model	Spearman's Correlation	p-value (2-tailed)	95% CI (2-tailed)
ChatGPT-4	-	-	-
Age - Time1	0.058	0.595	-0.157, 0.268
Age - Time2	-0.012	0.910	-0.225, 0.201
Age - Time3	0.012	0.911	-0.201, 0.224
Time1-Time2	0.752	<0.001	0.647, 0.829
Time1-Time3	0.629	<0.001	0.488, 0.738
Time2-Time3	0.911	<0.001	0.869, 0.940
Model	Spearman's Correlation	p-value (2-tailed)	95% CI (2-tailed)
ChatGPT o3-mini	-	-	-
Age - Time1	0.595	0.595	0.268, 0.938
Age - Time2	0.910	0.910	0.201, 0.981
Age - Time3	0.911	0.911	0.225, 0.971
Time1-Time2	0.991	<0.001	0.829, 0.991
Time1-Time3	0.987	<0.001	0.738, 0.924
Time2-Time3	0.998	<0.001	0.940, 0.983

Strong, significant correlations existed between Time variables (e.g., Time1-Time2, Time1-Time3, Time2-Time3), indicating strong internal consistency in the dataset. Minor differences in confidence intervals existed but were negligible.

ChatGPT o3-mini showed highly inaccurate results. Age-Time correlations were dramatically inflated compared to SPSS and ChatGPT-4, with values like 0.595, 0.910, and 0.911 instead of close-to-zero values. The p-values for Age-Time correlations are clearly incorrect (e.g., 0.595 for a correlation of 0.595, which is inconsistent with standard statistical interpretation).

Time1-Time2, Time1-Time3, and Time2-Time3 correlations were unrealistically high (0.991, 0.987, 0.998) compared to 0.752-0.911 in SPSS and ChatGPT-4. In terms of comparison across models, the SPSS vs. ChatGPT-4 results were statistically equivalent, with only minor differences in confidence intervals. ChatGPT o3-mini’s results were unreliable, with inflated correlations and incorrect p-values. Confidence intervals for ChatGPT o3-mini were also unrealistically narrow and shifted upward, suggesting systematic miscalculation.

SPSS and ChatGPT-4 provided accurate, comparable results for Spearman’s correlations. ChatGPT o3-mini’s results were flawed and should not be trusted for this analysis. Users should verify outputs with SPSS or ChatGPT-4 when working with correlation analyses.

Table 7 below presents simple linear regression results predicting Time3 (Dependent Variable) from Time2 (Independent Variable) across SPSS, ChatGPT-4, and ChatGPT o3-mini. The model fit was identical across all methods. The correlation coefficient (R = 0.977) indicated a very strong positive relationship between Time2 and Time3. The coefficient of determination (R² = 0.955) implies that 95.5% of the variance in Time3 is explained by Time2, confirming a very strong predictive model. Adjusted R² (0.955) remained the same, confirming model stability after, in this instance, no adjustment.

**Table 7:** Simple Linear Regression SEE=Standard Error of the Estimate, SE=Standard error of the Mean, β=Beta coefficient Simple linear regression was used as the statistical test to compute the p-values for this table.

Model	R	R^2^	Adjusted R^2^	SEE	F	p-value (2-tailed)	t	p-value	B	SE	β
SPSS	0.977	0.955	0.955	2.68562	1943.94	<0.001	44.09	<0.001	1.035	0.023	0.977
ChatGPT-4	0.977	0.955	0.955	2.68562	1943.94	<0.001	44.06	<0.001	0.985	0.022	0.977
ChatGPT o3-mini	0.977	0.955	0.955	2.68562	1943.94	<0.001	44.06	<0.001	0.985	0.022	0.977

The F-statistic (1943.94, p < 0.001) confirmed that the regression model was significant. The Standard Error of the Estimate (2.68562) was identical across all models, meaning the error margin in predictions was consistent. The t-values (~44.06-44.09, p < 0.001) confirmed that Time2 was a significant predictor of Time3. The unstandardized coefficient (B) varied slightly, SPSS: B=1.035, and ChatGPT-4 and o3-mini: B=0.985. The standardized coefficient (β = 0.977) was identical across all models, indicating that Time2 strongly predicted Time3 regardless of calculation variations.

In terms of comparison across models, i.e. SPSS vs. ChatGPT-4 and ChatGPT o3-mini, the overall model statistics (R, R², Adjusted R², F-statistic, p-values) were identical across all three. The only difference was in B (unstandardized coefficient), where SPSS reported 1.035, while ChatGPT models reported 0.985. This discrepancy was minor and likely due to computational rounding or different handling of decimal precision.

SPSS, ChatGPT-4, and ChatGPT o3-mini produced nearly identical regression results, reinforcing model reliability. The small difference in B-values (1.035 vs. 0.985) was unlikely to affect interpretation significantly, but SPSS is the most trusted source for precise statistical output. Overall, Time2 is a very strong predictor of Time3, with the regression model explaining over 95% of the variance.

Table 8 below presents the results of a One-Sample t-test comparing the sample mean of Time1 to a hypothetical population mean (μ) across SPSS, ChatGPT-4, and ChatGPT o3-mini. Identical results were reported across all methods. With a sample size n = 93, mean of time1=5.3226, SD=8.97601, standard error (SE)=0.93077, t-value=5.718, degrees of freedom (df)=92, p-value: <0.001, (statistically significant), mean difference=5.32258, confidence interval (95% CI) is (3.47, 7.17), effect size (Cohen’s d)=0.593, indicating a moderate effect size, effect size 95% CI indicated minor differences in ChatGPT models (0.372, 0.814) vs. SPSS (0.371, 0.812), likely due to rounding.

**Table 8:** One Sample t-test SD=Standard Deviation, CI=Confidence Interval, SE=Standard Error of the Mean, df=degrees of freedom, ES=Effect Size, 95%CI=95% Confidence Interval The One Sample t-test was used as the statistical test to compute the p-values for this table.

Model	n	Mean	SD	SE	t	df	p-value	Mean Difference	95%CI for Mean Difference	ES (Cohen's d)	ES 95%CI
SPSS	93	5.3226	8.97601	0.93077	5.718	92	<0.001	5.32258	3.47, 7.17	0.593	0.371, 0.812
ChatGPT-4	93	5.3226	8.97601	0.93077	5.718	92	<0.001	5.32258	3.47, 7.17	0.593	0.372, 0.814
ChatGPT o3-mini	93	5.3226	8.97601	0.93077	5.718	92	<0.001	5.32258	3.47, 7.17	0.593	0.372, 0.814

In terms of statistical significance and interpretation, the p-value (<0.001) indicated that the mean of Time1 was significantly different from the population mean (µ). The moderate effect size (Cohen’s d = 0.593) suggested a meaningful but not large deviation from µ.

SPSS vs. ChatGPT-4 and ChatGPT o3-mini results were practically identical, with only negligible rounding differences in Cohen’s d confidence intervals. All models confirmed that the difference was statistically significant with a moderate effect size. All three methods yielded the same t-test results, indicating strong consistency in calculations. Time1's mean was significantly different from the hypothesized population mean, with a moderate effect size. SPSS remains the gold standard, but ChatGPT-4 and ChatGPT o3-mini provide highly reliable results for this test.

Table 9 below presents the results of a paired t-test comparing pretest (Time1) and posttest (Time3) scores across SPSS, ChatGPT-4, and ChatGPT o3-mini. Identical results were found, across all methods, with the pretest mean (Time1)=5.3226, and the posttest mean (Time3)=11.2903, SD for pretest=8.97601, SD for posttest=12.63069, SE=0.93077 for the pretest, and 1.30974 for the posttest.

**Table 9:** Paired t-test SD=Standard Deviation, CI=Confidence Interval, SE=Standard Error of the Mean, df=degrees of freedom, ES=Effect Size, 95%CI=95% Confidence Interval The Paired t-test was used as the statistical test to compute the p-values for this table.

Model	Mean	SD	SE	t	df	p-value	Mean Difference	95%CI	Effect size (Cohen's d)	Effect size 95%CI
SPSS	-	-	-	-	-	-	-	-	-	-
Pretest Mean-Time1	5.3226	8.97601	0.93077	-7.939	92	<0.001	-5.96774	-7.46073, -4.47475	-0.823	-1.057, -0.586
Posttest Mean-Time3	11.2903	12.63069	1.30974	-	-	-	-	-	-	-
ChatGPT-4	-	-	-	-	-	-	-	-	-	-
Pretest Mean-Time1	5.3226	8.97601	0.93077	-7.939	92	<0.001	-5.96774	-7.46073, -4.47475	-0.823	-1.058, -0.588
Posttest Mean-Time3	11.2903	12.63069	1.30974	-	-	-	-	-	-	-
ChatGPT o3-mini	-	-	-	-	-	-	-	-	-	-
Pretest Mean-Time1	5.3226	8.97601	0.93077	-7.939	92	<0.001	-5.96774	-7.46073, -4.47475	-0.823	-1.058, -0.588
Posttest Mean-Time3	11.2903	12.63069	1.30974	-	-	-	-	-	-	-
-	-	-	-	-	-	-	-	-	-	-

The t-value was equal to -7.939, with the df=92, and the p-value <0.001 (statistically significant). The mean difference was -5.96774, with a 95% confidence interval (CI) for mean difference: (-7.46073, -4.47475). The effect size (Cohen’s d) was -0.823 which was a large effect with the effect size 95% CI with minor rounding differences across models (-1.057, -0.586 in SPSS vs. -1.058, -0.588 in ChatGPT models).

The p-value (<0.001) confirmed a significant difference between Pretest (Time1) and Posttest (Time3), suggesting a meaningful change over time. The negative t-value (-7.939) indicates that Time3 (Posttest) was significantly higher than Time1 (Pretest). The effect size (Cohen’s d = -0.823) suggested a large effect, indicating a strong difference between pretest and posttest scores.

With SPSS vs. ChatGPT-4 and ChatGPT o3-mini, the results were identical across all three models, with only tiny rounding differences in effect size confidence intervals. This confirmed the high accuracy of ChatGPT models when performing paired t-tests. All three models produced the same paired t-test results, confirming their reliability. SPSS remained the benchmark, but ChatGPT-4 and ChatGPT o3-mini provided equally valid results.

Table 10 below presents the results of a two-independent sample t-test comparing the means of Group 1 and Group 2 across SPSS, ChatGPT-4, and ChatGPT o3-mini. Descriptive statistics were identical across all methods, i.e. Group 1: Sample size (n)= 68 mean=11.2059, SD=11.82151, SE=1.43357. For Group 2: the sample size was n=22, with the mean equal to 12.2727, SD=15.52013, and the SE equal to 3.3089.

**Table 10:** Two-Independent Sample t-test SD=Standard Deviation, CI=Confidence Interval, SE=Standard Error of the Mean, df=degrees of freedom, ES=Effect Size, 95%CI=95% Confidence Interval The Two-Independent Sample t-test was used as the statistical test to compute the p-values for this table.

Model	n	Mean	SD	SE	t	df	p-value (2-tailed)	Mean Difference	95%CI	ES (Cohen's d)	ES (95%CI)
SPSS	-	-	-	-	-	-	-	-	-	-	-
Group 1 Mean	68	11.206	11.822	1.434	-0.340	88	0.735	-1.067	-7.307, 5.173	-0.083	-0.564, 0.398
Group 2 Mean	22	12.273	15.520	3.309	-	-	-	-	-	-	-
ChatGPT-4	-	-	-	-	-	-	-	-	-	-	-
Group 1 Mean	68	11.206	11.822	1.434	-0.296	29.3	0.770	-1.067	-8.439, 6.305	-0.083	-0.564, 0.398
Group 2 Mean	22	12.273	15.520	3.309	-	-	-	-	-	-	-
ChatGPT o3-mini	-	-	-	-	-	-	-	-	-	-	-
Group 1 Mean	68	11.206	11.821	1.434	-0.296	29	0.770	-1.067	-8.439, 6.305	-0.083	-0.564, 0.398
Group 2 Mean	22	12.273	15.520	3.309	-	-	-	-	-	-	-

The two-independent sample t-test results differed slightly between SPSS and ChatGPT Models. With SPSS, the t-value was -0.34, with df at 88, a p-value of 0.735, with a 95% CI for mean difference at (-7.30678, 5.17309).

With ChatGPT-4 and ChatGPT o3-mini, the t-value was -0.296 (slight difference from SPSS), with df: ~29 (lower than SPSS due to a different df calculation method), p-value equal to 0.77 (slightly different from SPSS), with a 95% CI for mean difference: (-8.4388, 6.3052). The effect size (Cohen’s d) was identical across all models with Cohen’s d equal to -0.083, and effect size 95% CI: (-0.564, 0.398). This suggested a very small, negligible effect size, meaning there is little to no meaningful difference between Group 1 and Group 2.

With SPSS vs. ChatGPT-4 and ChatGPT o3-mini, the descriptive statistics (means, SDs, SEs) were identical across all three models. SPSS used a standard pooled variance approach for degrees of freedom (df = 88), while ChatGPT-4 and ChatGPT o3-mini used Welch’s t-test, which adjusts df for unequal variances (df ≈ 29). The t-values (-0.34 vs. -0.296) and p-values (0.735 vs. 0.77) differed slightly due to this difference in df calculation. The effect size (Cohen’s d) was identical, confirming that the overall conclusion remained unchanged.

All three models showed no significant difference between Group 1 and Group 2 (p > 0.05). SPSS and ChatGPT-4/ChatGPT o3-mini used different t-test calculations (pooled variance vs. Welch’s t-test), leading to small differences in t-values and p-values. Effect size (Cohen’s d) confirmed a negligible difference between the groups. If the assumption of equal variances holds, SPSS’s results are preferred; if variances are unequal, ChatGPT-4 and ChatGPT o3-mini’s results (Welch’s t-test) are more appropriate.

Advanced statistics

Table 11 below presents the results of a multiple linear regression analysis predicting Time3 (Dependent Variable, DV) from Time2, Time1, and Age (Independent Variables, IVs) across SPSS, ChatGPT-4, and ChatGPT o3-mini. In terms of model fit differences for R, R², and adjusted R², with SPSS: R = 0.985, which indicated a very strong relationship between predictors (Age, Time1, Time2) and Time3. The R² equal to 0.971 implied that 97.1% of the variance in Time3 was explained by the predictors. With the adjusted R² equal to 0.970, the model remained strong even after adjusting for the number of predictors.

**Table 11:** Multiple Linear Regression SEE=Standard Error of the Estimate, SE=Standard Error of the Mean Multiple Llnear Regression was used as the statistical test to compute the p-values for this table.

Model	R	R^2^	Adjusted R^2^	SEE	F	p-value	t	p-value	B	SE	β
SPSS	0.985	0.971	0.970	2.263	895.624	<0.001	-	-	-	-	-
Age	-	-	-	-	-	-	-0.533	0.595	-0.127	0.238	-0.010
Time1	-	-	-	-	-	-	-2.645	0.010	-0.146	0.055	-0.104
Time2	-	-	-	-	-	-	27.649	<0.001	1.141	0.041	1.076
ChatGPT-4	0.979	0.958	0.957	2.646	668.34	<0.001	-	-	-	-	-
Age	-	-	-	-	-	-	-0.343	0.732	-0.012	0.034	-0.343
Time1	-	-	-	-	-	-	-2.320	0.023	-0.146	0.063	-2.318
Time2	-	-	-	-	-	-	23.73	<0.001	1.132	0.048	23.730
ChatGPT o3-mini	0.979	0.958	0.957	2.646	668.340	<0.001	-	-	-	-	-
Age	-	-	-	-	-	-	0.138	0.890	0.012	0.087	0.015
Time1	-	-	-	-	-	-	-0.486	0.627	-0.054	0.111	-0.063
Time2	-	-	-	-	-	-	44.909	<0.001	0.987	0.022	0.977

With ChatGPT-4 and ChatGPT o3-mini, R = 0.979 (slightly lower than SPSS), with R² equal to 0.958 (lower than SPSS, meaning 95.8% of variance explained). The adjusted R² = 0.957 (slightly lower than SPSS). SPSS suggested a stronger overall model fit than ChatGPT-4 and ChatGPT o3-mini.

In terms of statistical significance of predictors, with SPSS, Time2 (B = 27.649, p < 0.001) was the strongest predictor with a large positive effect. Time1 (B = -2.645, p = 0.01) was statistically significant but had a smaller negative effect. Age (B = -0.533, p = 0.595) was not significant (p > 0.05), meaning Age did not contribute much to predicting Time3.

With ChatGPT-4, Time2 (B = 23.73, p < 0.001) remained the strongest predictor, but its coefficient was smaller than in SPSS. Time1 (B = -2.32, p = 0.023) was statistically significant but weaker than in SPSS. Age (B = -0.343, p = 0.732) was not significant and had a weaker effect than in SPSS.

With the ChatGPT o3-mini, Time2 (B = 44.909, p < 0.001) was the strongest predictor, but its coefficient was much higher than in SPSS and ChatGPT-4. Time1 (B = -0.486, p = 0.627) was not significant, unlike in SPSS and ChatGPT-4. And Age (B = 0.138, p = 0.89) was not significant and had an opposite direction from SPSS and ChatGPT-4.

SPSS and ChatGPT-4 provided similar significance patterns, though SPSS suggested a stronger model. ChatGPT o3-mini's coefficients were inconsistent, especially for Time2, which is unrealistically inflated (B = 44.909 vs. SPSS’s B = 27.649).

In terms of model performance (F-statistic and standard error of the estimate), SPSS (F = 895.624, p < 0.001) suggested a highly significant model. ChatGPT-4 and o3-mini (F = 668.34, p < 0.001) had lower F-values, meaning a weaker overall model fit compared to SPSS. With the standard error of the estimate (SEE), the SPSS result is 2.26277 (lower, indicating better fit). With ChatGPT-4 and o3-mini, the SEE was 2.646 (higher, indicating slightly more error in predictions).

SPSS outperformed ChatGPT models in model fit and provided a more reliable multiple regression analysis. SPSS provided the strongest model, with the highest R², lowest standard error, and best predictor significance. ChatGPT-4 follows closely but underestimates Time2's effect compared to SPSS. ChatGPT o3-mini had large inconsistencies in coefficients, particularly for Time2, making its results less reliable.

SPSS was the most reliable source for this multiple regression analysis. ChatGPT-4 was similar but produced slightly weaker model performance. ChatGPT o3-mini showed inconsistent coefficient values, particularly an overestimated Time2 effect, making its results questionable. Overall, Time2 was the strongest predictor of Time3, while Age was not a significant predictor in any model.

Table 12 below presents the results of a one-way ANOVA analyzing the effect of Age Group (IV) on Time3 (DV) across SPSS, ChatGPT-4, and ChatGPT o3-mini. There were identical results across all models (SPSS, ChatGPT-4, ChatGPT o3-mini).

**Table 12:** One-Way Analysis of Variance (ANOVA) df=degrees of freedom One-Way Analysis of Variance (ANOVA) was used as the statistical test to compute the p-values for this table.

Source of Variation	Sum of Squares	df	Mean Square	F	p-value
SPSS	-	-	-	-	-
Between Groups	648.112	4	162.028	0.959	0.435
Within Groups	13517.464	80	168.968	-	-
Total	14165.576	84	-	-	-
ChatGPT-4	-	-	-	-	-
Source of Variation	Sum of Squares	df	Mean Square	F	p-value
Between Groups	648.112	4	162.028	0.959	0.435
Within Groups	13517.464	80	168.968	-	-
Total	14165.576	84	-	-	-
ChatGPT o3-mini	-	-	-	-	-
Source of Variation	Sum of Squares	df	Mean Square	F	p-value
Between Groups	648.112	4	162.028	0.959	0.435
Within Groups	13517.464	80	168.968	-	-
Total	14165.576	84	-	-	-

Sum of squares (between groups)=648.112, df for between groups is 4, mean square (between groups) was equal to 162.028. The sum of squares (within groups) is 13,517.464, with df for within groups was equal to 80. The mean square (within groups) is 168.968, with the total sum of squares was equal to 14,165.576. The F-statistic was 0.959, with the p-value equal to 0.435.

The F-value (0.959) was low, suggesting little variance in Time3 explained by Age Group. The p-value (0.435) was greater than 0.05, indicating no statistically significant difference in Time3 between different Age Groups. Since Age Group did not significantly impact Time3, the groups were statistically similar in their Time3 scores.

SPSS, ChatGPT-4, and ChatGPT o3-mini produced exactly the same results. There were no differences in calculations or rounding. This confirmed that ChatGPT models were fully aligned with SPSS in performing one-way ANOVA. Age Group did not have a statistically significant effect on Time3 (p = 0.435). No post-hoc analysis was conducted as the results were non-significant.

Since all three models yielded identical results, ChatGPT-4 and ChatGPT o3-mini can be reliably used for ANOVA tests. SPSS remains the gold standard, but ChatGPT models produce accurate, reproducible results for this analysis.

Table 13 below presents the results of a repeated measures ANOVA comparing SPSS, ChatGPT-4, and ChatGPT o3-mini in assessing the effect of Time on a dependent variable. With the SPSS results, we had a Significant Main Effect of Time with F(2, 184) = 55.701, p < 0.001, which indicated a statistically significant difference across time points.

**Table 13:** Repeated Measures Analysis of Variance (ANOVA) df=degrees of freedom

Source of Variation		Type III Sum of Squares	df	Mean Square	F	p-value							
SPSS	-	-	-	-	-	-	-	-	-	-	-	-	-
Time	Sphericity Assumed	1780.007	2	890.004	55.701	<0.001	-	-	-	-	-	-	-
-	Greenhouse-Geisser (GG)	1780.007	1.188	1498.847	55.701	<0.001	-	-	-	-	-	-	-
-	Huynh-Feldt (HF)	1780.007	1.194	1490.582	55.701	<0.001	-	-	-	-	-	-	-
-	Lower-bound (LB)	1780.007	1	1780.007	55.701	<0.001	-	-	-	-	-	-	-
Error (Time)	Sphericity Assumed	2939.993	184	15.978	-	-	-	-	-	-	-	-	-
-	Greenhouse-Geisser (GG)	2939.993	109.258	26.909	-	-	-	-	-	-	-	-	-
-	Huynh-Feldt (HF)	2939.993	109.864	26.760	-	-	-	-	-	-	-	-	-
-	Lower-bound (LB)	2939.993	92	31.956	-	-	-	-	-	-	-	-	-
ChatGPT-4	-	-	-	-	-	-	-	-	-	-	-	-	-
-	F Value	Numerator df	Denominator df	p-value	-	-	-	-	-	-	-	-	-
Time	55.70104	2	184	1.22E-19	-	-	-	-	-	-	-	-	-
-	-	-	-	-	-	-	-	-	-	-	-	-	-
ChatGPT o3-mini	-	-	-	-	-	-	-	-	-	-	-	-	-
Source	F Value	Numerator df (Sphericity Assumed)	Denominator df (Sphericity Assumed)	p (Sphericity Assumed)	Numerator df (GG)	Denominator df (GG)	p (GG)	Numerator df (HF)	Denominator df (HF)	p (HF)	Numerator df (LB)	Denominator df (LB)	p (LB)
Time	55.7	2	184	1.22E-19	1.56	143.5	2.50E-18	1.78	164	8.00E-19	1	92	1.05E-16

In terms of Sphericity Assumption and Corrections, with Sphericity Assumed, F=55.701, p<0.001. With the Greenhouse-Geisser (GG) Correction, F=55.701, p<0.001, adjusted df=1.188. The Huynh-Feldt (HF) Correction produced an F=55.701, p<0.001, F = 55.701, p < 0.001, adjusted df = 1.194. The Lower-bound (LB) Correction again produced an F=55.701, p<0.001, with an adjusted df of 1.

The error terms differed based on the correction used were Sphericity Assumed: SS = 2939.993, df = 184, MS = 15.978; Greenhouse-Geisser: df = 109.258, MS = 26.909, Huynh-Feldt: df = 109.864, MS = 26.76, Lower-bound: df = 92 with an MS of 31.956.

The ChatGPT-4 results reported only the main effect: F(2,184) = 55.70104, p = 1.22E-19, which was equivalent to SPSS.

No sphericity corrections were provided, meaning it assumed sphericity was met.

The ChatGPT o3-mini results included sphericity corrections, similar to SPSS: Sphericity Assumed: F=55.7, p=1.22E−19F = 55.7, p = 1.22E-19 (consistent with SPSS). Greenhouse-Geisser: df = 1.56, 143.5, p = 2.50E-18. Huynh-Feldt: df = 1.78, 164, p = 8.00E-19. Lower-bound: df = (1, 92), p = 1.05E-16. Slight rounding differences existed but results aligned closely with SPSS.

SPSS and ChatGPT o3-mini provided detailed sphericity adjustments, whereas ChatGPT-4 only reported the main effect. All three models showed a significant effect of Time (p < 0.001), confirming differences across time points. ChatGPT-4 was accurate for basic analysis but lacked the sphericity corrections needed for a more robust interpretation. ChatGPT o3-mini closely aligned with SPSS, making it more reliable than ChatGPT-4 for this analysis.

Time significantly affected the dependent variable across all models. SPSS and ChatGPT o3-mini provided full sphericity adjustments, making them preferable for in-depth analysis. ChatGPT-4 was useful for quick results but did not account for sphericity violations. If sphericity is a concern, corrections from SPSS or ChatGPT o3-mini should be used.

Table 14 below presents the results of a two-way (factorial) ANOVA analyzing the effects of Gender and AgeCategory (Independent Variables) on a Dependent Variable across SPSS, ChatGPT-4, and ChatGPT o3-mini. With the SPSS results, there were no significant main effects or interaction effects: Gender (F = 0.473, p = 0.494) was not significant. AgeCategory (F = 1.024, p = 0.400) was not significant. Gender × AgeCategory interaction (F = 0.48, p = 0.750) was not significant. In terms of overall model fit, the adjusted R² = -0.042, indicating the model did not explain much variation in the dependent variable.

**Table 14:** Two-Way (Factorial) Analysis of Variance (ANOVA) Two-Way (Factorial) ANOVA was used as the statistical test to compute the p-values for this table. df=degrees of freedom

Source of Variation	Sum of Squares	df	Mean Square	F	p-value
SPSS	-	-	-	-	-
Corrected Model	987.077	9	109.675	0.624	0.773
Intercept	5595.019	1	5595.019	31.842	<0.001
Gender	83.138	1	83.138	0.473	0.494
AgeCategory	719.969	4	179.992	1.024	0.4
Gender * AgeCategory	337.321	4	84.33	0.48	0.75
Error	13178.499	75	175.713	-	-
Total	26143	85	-	-	-
Corrected Total	14165.576	84	-	-	-
Adjusted R-Squared	-0.042	-	-	-	-
ChatGPT-4	-	-	-	-	-
Source of Variation	Sum of Squares	df	Mean Square	F	p-value
Intercept	1764	1	1764	10.03907926	0.002218
Gender	32.267	1	32.267	0.184	0.67
AgeCategory	610.476	4	152.619	0.869	0.487
Gender * AgeCategory	337.321	4	84.33	0.478	0.75
Error(Time)	13178.4994	75	175.7133254	-	-
Adjusted R-Squared	-0.042	-	-	-	-
ChatGPT o3-mini	-	-	-	-	-
Source of Variation	Sum of Squares	df	Mean Square	F	p-value
Corrected Model	12964.501	10	1296.45	7.378	< 0.001
Intercept	5595.019	1	5595.019	31.842	<0.001
Gender	83.138	1	83.138	0.473	0.494
AgeCategory	719.969	4	179.992	1.024	0.4
Gender × AgeCategory	337.321	4	84.33	0.48	0.75
Error	13178.499	75	175.713	-	-
Total	26143	85	-	-	-
Corrected Total	14165.576	84	-	-	-
Adjusted R Squared	0.429	-	-	-	-

With the ChatGPT-4 results, there were slight discrepancies in sum of squares for AgeCategory (610.48 vs. 719.97 in SPSS). The Gender × AgeCategory interaction matches SPSS exactly (F = 0.48, p = 0.75). The adjusted R² (-0.04196) closely matched SPSS (-0.042), confirming similar conclusions. The statistical interpretation aligned with SPSS, showing no significant effects.

With the ChatGPT o3-mini results, there were major discrepancies in model sum of squares (corrected model = 12,964.501 vs. 987.077 in SPSS). The reported R² (0.496) and adjusted R² (0.429) were unrealistic given the SPSS and ChatGPT-4 results. While individual factor effects (Gender, AgeCategory, and their interaction) matched SPSS, the inflated overall model statistics suggested a calculation error. This made ChatGPT o3-mini's results unreliable for this analysis.

Table 15 below presents the results of a MANOVA comparing SPSS, ChatGPT-4, and ChatGPT o3-mini, which examined the effect of Gender on multiple dependent variables. With the SPSS results, the intercept was highly significant across all tests (p < 0.001, Partial Eta Squared = 0.96), meaning the overall model explained a large proportion of variance.

**Table 15:** Multivariate Analysis of Variance (MANOVA) Multivariate Analysis of variance (MANOVA) was used as the statistical test to compute the p-values for this table. df=degrees of freedom

Source of Variation		Value	F	Hypothesis df	Error df	Sig.	Partial Eta Squared
SPSS	Test	-	-	-	-	-	-
Intercept	Pillai's Trace	0.960	270.045	8	90	<0.001	0.96
-	Wilks' Lambda	0.040	270.045	8	90	<0.001	0.96
-	Hotelling's Trace	24.004	270.045	8	90	<0.001	0.96
-	Roy's Largest Root	24.004	270.045	8	90	<0.001	0.96
Gender	Pillai's Trace	0.082	1.001	8	90	0.441	0.082
-	Wilks' Lambda	0.918	1.001	8	90	0.441	0.082
-	Hotelling's Trace	0.089	1.001	8	90	0.441	0.082
-	Roy's Largest Root	0.089	1.001	8	90	0.441	0.082
ChatGPT-4	-	-	-	-	-	-	-
-	Test	Value	Num DF	Den DF	F Value	p-value	-
-	Wilks' Lambda	0.7295	8	10	0.4635	0.8559	-
-	Pillai’s Trace	0.2705	8	10	0.4635	0.8559	-
-	Hotelling-Lawley Trace	0.3708	8	10	0.4635	0.8559	-
-	Roy’s Largest Root	0.3708	8	10	0.4635	0.8559	-
ChatGPT o3-mini	-	-	-	-	-	-	-
-	Test	Value	Num DF	Den DF	F Value	p-value	-
-	Wilks' Lambda	0.7295	8	10	0.4635	0.8559	-
-	Pillai’s Trace	0.2705	8	10	0.4635	0.8559	-
-	Hotelling-Lawley Trace	0.3708	8	10	0.4635	0.8559	-
-	Roy’s Largest Root	0.3708	8	10	0.4635	0.8559	-

Gender was not a significant factor (p = 0.441 across all multivariate tests), which suggested no meaningful effect on the dependent variables. All four multivariate tests (Pillai's Trace, Wilks’ Lambda, Hotelling’s Trace, and Roy’s Largest Root) showed similar F-values (1.001) and low effect sizes (Partial Eta Squared = 0.082), reinforcing the non-significant effect of Gender with the SPSS results.

With the ChatGPT-4 and ChatGPT o3-mini results, we had incorrect df. SPSS results indicate that error df = 90, while both ChatGPT models indicated that the error df = 10, which suggests ChatGPT models miscalculated the error degrees of freedom. The ChatGPT-4 and ChatGPT o3-mini F-values and p-values differed notably from SPSS, with the SPSS' F = 1.001, with a p = 0.441.

With the ChatGPT models, we had an F = 0.4635, with a p equal to 0.8559. The p-values in ChatGPT models were much higher (0.8559 vs. 0.441 in SPSS), which suggested a miscalculation in variance estimation. Wilks’ Lambda (SPSS = 0.918, ChatGPT models = 0.7295). A notable discrepancy in Wilks’ Lambda values suggested that ChatGPT models may have applied different computational assumptions.

SPSS provided the most reliable MANOVA results, showing that Gender was not a significant predictor (p = 0.441). ChatGPT-4 and ChatGPT o3-mini produced incorrect results due to errors in degrees of freedom, leading to inaccurate F-values and p-values. The ChatGPT models underestimated the significance of Gender (p = 0.8559 instead of 0.441), making their results unreliable for this analysis. For MANOVA, SPSS should be used as the primary source, as ChatGPT models failed to match its accuracy.

## Discussion

The increasing integration of AI into statistical analysis represents a significant shift in research methodologies across multiple disciplines, particularly in medical and epidemiological research. This study systematically compared the performance of SPSS, ChatGPT-4, and ChatGPT o3-mini in executing fundamental and advanced statistical procedures, contributing to the growing literature on AI-based statistical tools' reliability, accuracy, and consistency.

Performance of AI in fundamental statistical analyses

Findings indicate high concordance between SPSS and ChatGPT-4 in performing fundamental descriptive and inferential statistics, including measures of central tendency and dispersion (Table 1). The strong alignment in Pearson and Spearman correlation analyses between SPSS and ChatGPT-4 further supports its reliability in preliminary data exploration (Tables 2, 3). These results align with previous findings by Shahrul and Mohamed [6], suggesting that ChatGPT-4 is a viable tool for routine statistical computations.

However, ChatGPT o3-mini exhibited discrepancies in multiple correlational analyses, producing inflated values and spurious statistical significance (Table 5). Such inconsistencies raise concerns about its adherence to established mathematical conventions, particularly in hypothesis-driven research where statistical accuracy is crucial. Given the impact of statistical miscalculations on effect size interpretation and hypothesis validation, researchers must exercise caution when relying on ChatGPT o3-mini for statistical inference, particularly in fields where statistical rigor informs clinical and policy decisions [6].

AI performance in t-tests, regression, and complex statistical models

ChatGPT-4 demonstrated strong consistency with SPSS across various inferential tests, including t-tests and simple linear regression (Tables 6-8). This supports its applicability in biomedical and social sciences, where mean comparisons and effect sizes are critical [6,8]. However, discrepancies emerged when more complex statistical techniques were examined. ChatGPT o3-mini produced inflated coefficients and R² values in two-way ANOVA and multiple regression models (Tables 10, 13), suggesting deviations in its computational assumptions. These errors can lead to misinterpretations in studies assessing variable interactions and predictor importance, particularly concerning applied research domains such as epidemiology, finance, and psychology [6,8,14,15].

Multiple regression analysis is commonly used to determine predictor significance, and inflated R² values may falsely suggest more substantial explanatory power, leading to misleading conclusions. Given that traditional statistical software like SPSS employs transparent computational assumptions and collinearity adjustments, ChatGPT o3-mini’s internal mechanisms require further scrutiny to ensure accuracy and reproducibility in complex model applications [14,15].

AI in multivariate and advanced statistical procedures

While ChatGPT-4 performed reliably in one-way ANOVA (Table 11), inconsistencies emerged in its execution of multivariate analyses. MANOVA computations resulted in miscalculated degrees of freedom, affecting significance values (Table 14). Given that MANOVA extends ANOVA by incorporating multiple dependent variables, precision in covariance structure computation is crucial. These findings align with earlier concerns regarding AI’s difficulty handling statistical dependencies in multivariate frameworks [9]. Such limitations highlight the need for further refinement in AI-driven multivariate testing, particularly in fields where simultaneous assessment of multiple outcomes is common.

Further errors were identified in factorial ANOVA, where ChatGPT o3-mini produced erroneous F-statistics when analyzing multiple independent variables [20-22]. Factorial ANOVA is essential for evaluating interaction effects, and incorrect variance partitioning could lead to misleading conclusions in experimental research. Similarly, ChatGPT-4 struggled with repeated measures ANOVA, failing to implement sphericity corrections (e.g., Greenhouse-Geisser or Huynh-Feldt adjustments), which could lead to inflated F-statistics and underestimated p-values. Given the significance of assumption checks in these analyses, AI-driven statistical tools must integrate built-in assumption diagnostics to ensure reliable interpretations [14,15].

Implications for AI adoption in statistical analysis

Despite these limitations, AI-driven statistical tools present transformative opportunities for automating data analysis and enhancing accessibility for non-statisticians. Meo et al. demonstrated that ChatGPT-4 achieved over 80% accuracy in statistical problem-solving, underscoring its potential as a pedagogical tool for teaching research methodologies. AI-based statistical analysis offers real-time feedback, automated computations, and natural language-based interactions, reducing entry barriers for students unfamiliar with statistical software such as SPSS, R, or SAS [1].

However, the observed discrepancies in complex statistical models highlight the importance of cross-validation with established tools. Traditional statistical software follows standardized methodologies, allowing researchers to verify and diagnose errors systematically. Since AI models often function as black-box systems with opaque internal computations, ensuring computational transparency is essential for scientific reproducibility [6,8].

AI model optimization needs

Future developments should enhance computational transparency to improve AI's applicability in statistical research. AI-driven models should provide detailed methodological documentation akin to SPSS, R, and SAS. Integrating assumption checks and corrections with built-in diagnostics for normality, homoscedasticity, and collinearity should be incorporated into AI models to improve reliability [6]. Refining variance partitioning techniques will optimize multivariate computation and error term adjustments in multivariate models such as MANOVA and repeated measures ANOVA. Facilitating interoperability with statistical programming languages, such as direct application programming interface (API) integrations with R, Python, and SAS, could improve AI accuracy by leveraging trusted statistical libraries [6].

While AI models like ChatGPT-4 perform well in structured statistical problem-solving, inconsistencies in complex statistical techniques necessitate continued validation against traditional statistical software. AI's role in statistical analysis will likely expand, but ensuring accuracy, transparency, and methodological alignment with conventional statistical standards remains paramount for its widespread adoption in scientific research [6,8].

Limitations and future directions

This research has several limitations. This comparative analysis was conducted using pre-formatted datasets originally optimized for SPSS, which may not accurately reflect the performance of AI models when applied to raw, unstructured, or inconsistently formatted data. Although prompt consistency was maintained, this constraint may restrict the broader applicability of the findings. To bridge this limitation, future investigations should assess the ability of AI systems to handle a more diverse range of data formats. This includes evaluating their performance in essential data management tasks such as preprocessing, data cleaning, and the imputation of missing values, common and often complex challenges encountered in real-world datasets.

This study did not assess the interoperability of AI models with widely used statistical programming environments such as R, SAS, STATA, or Python. Given that many researchers validate their analyses across multiple software platforms to ensure consistency, understanding how AI-based tools integrate with these statistical ecosystems is vital. Such integration is essential for facilitating seamless workflows and enhancing the practical utility of AI in multidisciplinary research settings.

Although our study focused on foundational inferential data analyses, it did not investigate the application of advanced statistical techniques. Methodologies such as hierarchical linear modeling, Bayesian inference, Principal Components Analysis (PCA), and machine learning-based statistical approaches are increasingly prevalent in contemporary medical research, particularly in fields requiring complex data structures or probabilistic modeling. Evaluating AI models’ competence in implementing and interpreting such sophisticated methodologies remains an important avenue for future inquiry, and we expect that such advancements will emerge as the technology matures.

A notable limitation pertains to the dependency of ChatGPT-4 on the specificity and clarity of user input. Unlike SPSS, which operates through standardized command structures and well-defined procedures, AI-generated outputs may be sensitive to variations in prompt construction and dataset descriptions. This raises concerns about the consistency and reliability of AI-based analysis when prompts are imprecise or ambiguous. Future work should explore strategies for prompt standardization or the development of structured input frameworks to minimize variability and enhance reproducibility in AI-assisted statistical reasoning.

Though not the impetus of this manuscript, sample representativeness and potential biases in data collection were not explicitly addressed in the context of this AI evaluation. The study did not assess the AI models' capabilities in error detection or their ability to perform real-time statistical diagnostics. Traditional statistical software often includes built-in tools for identifying violations of assumptions, outliers, and other issues that may compromise analytic validity. Current AI models generally lack explicit self-auditing or diagnostic functionalities. Enhancing future AI tools with automated mechanisms for assumption checking, error flagging, and data quality assessment will be essential for their adoption in high-stakes scientific research. In this comparative study, assumptions such as normality and reliability were not considered central to the validity of the findings. These assumptions typically pertain to specific statistical tests and the control of Type I error rates. However, the primary aim here was to evaluate the performance of AI-driven platforms relative to a traditional statistical software, not to conduct a conventional empirical analysis. This distinction enabled an objective comparison of platform capabilities, independent of standard statistical assumptions.

Addressing these limitations is crucial for refining AI-based statistical tools. Ensuring accuracy, reliability, and compatibility with established scientific methodologies will strengthen their role in evidence-based research and facilitate their integration into standard analytic practices.

## Conclusions

This study underscored the rapid advancements in AI-driven statistical analyses while highlighting areas that require further refinement. ChatGPT-4 accurately executed fundamental statistical tests, closely matching SPSS. However, its reliability diminished in more advanced statistical procedures, requiring further validation. ChatGPT o3-mini, while optimized for STEM applications, produced inconsistencies in correlation and multivariate analyses, limiting its dependability for complex research applications. Ensuring its alignment with established statistical methodologies will be essential for widespread scientific research adoption as AI evolves.
